# Bitter melon extract attenuating hepatic steatosis may be mediated by FGF21 and AMPK/Sirt1 signaling in mice

**DOI:** 10.1038/srep03142

**Published:** 2013-11-05

**Authors:** Yongmei Yu, Xian H. Zhang, Blake Ebersole, David Ribnicky, Zhong Q. Wang

**Affiliations:** 1 Nutrition and Diabetes Research Laboratory, Pennington Biomedical Research Center, LSU System. Baton Rouge, LA 70808; 2 Verdure Sciences, 1250 Conner St, Noblesville, IN 46060; 3 Dept. of Plant Biology and Path., Rutgers University, New Brunswick, New Jersey 08901

## Abstract

We sought to evaluate the effects of *Momordica charantia* (bitter melon, BM) extract on insulin sensitivity, NAFLD, hepatic FGF21 and AMPK signaling in mice fed a high-fat diet. Male C57/B6 mice were randomly divided into HFD and HFD supplementation with BM for 12 week. Body weight, plasma glucose, FGF21 and insulin levels, hepatic FGF21 and AMPK signaling proteins were measured. The results showed that plasma FGF21 and insulin concentrations were significantly decreased and hepatic FGF21 content was significantly down-regulated, while FGF receptors 1, 3 and 4 (FGFR1, FGFR3 and FGFR4) were greatly up-regulated in BM group compared to the HFD group (P < 0.05 and P < 0.01). BM also significantly increased hepatic AMPK p, AMPK α1 AMPK α2 and Sirt1 content compared to the HFD mice. We, for the first time, demonstrated that BM extract attenuated hepatic steatosis in mice by enhancing hepatic FGF21 and AMPK/Sirt1 signaling.

The prevalence of obesity, metabolic syndrome, and type 2 diabetes mellitus (T2DM) has increased to epidemic proportions worldwide^1^,^2^. Non-alcoholic fatty liver disease (NAFLD), which is associated with insulin resistance, oxidative stress, and inflammation, is also on the rise. Excess dietary fat intake is a risk factor for obesity and insulin resistance, which in turn contribute significantly to the development of T2DM and cardiovascular disease (CVD)^3^. High-fat diet (HFD) has also been linked to NAFLD, and a "lipotoxicity" hypothesis has been proposed suggesting that fat-induced hepatic insulin resistance may play a major role in the pathogenesis of type 2 diabetes^4^.

It is well documented that members of the fibroblast growth factor (FGF) family play numerous roles in cellular processes including growth, angiogenesis, and development^5^,^6^. FGFs modulate cellular activity via at least 5 distinct subfamilies of high-affinity FGF receptors (FGFRs): FGFR-1, -2, -3, -4, and -5^6^,^7^. Fibroblast growth factor 21 (FGF21) is an atypical member of the FGF family that functions as an endocrine hormone^5^,^8^ with broad metabolic actions in obese rodents and primates, which include enhancing insulin sensitivity, decreasing triglyceride (TG) concentrations, and reducing body weight. Circulating levels of FGF21 are strongly related to body weight and plasma levels of leptin, adiponectin, and insulin in normal-weight women^9^,^10^. In adipocytes, FGF21 induces glucose transporter-1 expression through activation of the serum response factor/E-twenty six-like protein-1(SRF/Elk-1)^11^ and regulates energy metabolism by activating the AMPK-SIRT1-PGC-1 alpha pathway^12^. FGF-21 has been identified as a novel metabolic regulator based on findings that it protects animals from diet-induced obesity when overexpressed in transgenic mice and lowers blood glucose and triglyceride levels when administered to diabetic rodents^13^,^14^. On the other hand, increased FGF21 levels were observed to correlate with elevated hepatic triglyceride content in NAFLD patients^15^. Thus, FGF 21 was suggested as a biomarker for NAFLD^16^. Moreover, newly diagnosed T2DM patients were found to have significantly higher plasma FGF-21 concentrations than nondiabetic control subjects, and elevated plasma FGF-21 levels have been observed in insulin-resistant states^14^,^17^. This is further supported by the findings that plasma FGF21 levels were significantly increased in subjects with prediabetes, diabetes and predicted the development of diabetes in humans^18^.

Bitter melon (BM), also called *Momordica charantia* is a popular fruit used for the treatment of diabetes and related conditions amongst the indigenous populations of Asia, South America, India, and East Africa. Several pre-clinical studies have documented the anti-diabetic and hypoglycemic effects of BM through various postulated mechanisms^19^. BM extracts have been reported to increase glucose uptake, promote insulin release, and potentiate the effect of insulin, as well as improve obesity-associated peripheral inflammation and neuroinflammation, lower plasma apoB-100 and apoB-48 in HFD-fed mice, and modulate the phosphorylation of IR, IRS-1, and its downstream signaling molecules^20^,^21^,^22^. Bioactive compounds of BM, cucurbitane triterpenoids, stimulate GLUT4 translocation to the cell membrane by activation of the AMPK pathway in both L6 myotubes and 3T3-L1 adipocytes^23^. Supplementation of BM to rats fed a high-fructose diet during gestation and lactation ameliorates fructose-induced dyslipidemia and hepatic oxidative stress in male offspring^1^. Our previous study shows that BM extract enhances insulin signaling, increases GLUT4 abundance and modulates acylcarnitine content in the skeletal muscle of HFD fed mice^24^. The precise mechanism by which BM extract improves glucose metabolism is largely unknown, and the effects of BM extracts on FGF21 signaling in obesity and metabolic syndrome have not been studied. Based on the above rationale, and given the role of FGF21 in lipid metabolism, we postulated that FGF21 may mediate the hypoglycemic action of BM. To test this hypothesis, we evaluated the effects of a BM extract on glucose and lipid metabolism, liver fat content, insulin sensitivity, and FGF21 signaling in mice fed a HFD.

## Results

**Figure 1:**
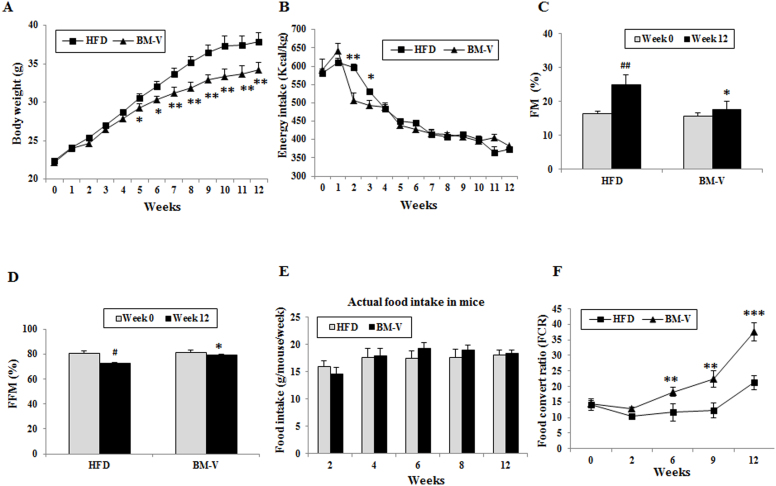
Effects of BM extract on body weight, food intake, body composition and FCR in mice fed a HFD. Two groups of mice were fed a HFD with or without BM-V for 12 weeks. Body weight and food intake are shown in panels (A) and (B), respectively. Body composition was measured as described in the methods. (C) Fat mass (FM) (% of body weight), (D) free fat mass (FFM) (% of body weight). (E) FCR results which were calculated as mass of the food eaten divided by the body mass gain over one week. The actual food intakes were presented as mean food intake/g/mouse/week (Fig. 1 F). Mean ± SEM (n = 10/group). Asterisk symbol stands for statistical comparison of BM-V group vs. HFD group; * P < 0.05, ** P < 0.01 and *** P < 0.001. # P < 0.05 and ## P < 0.01, Wk 0 vs. Wk12 in the HFD group.

Effects of BM extracts on body weight, food intake, body composition and Food convert ratio (FCR) in the HFD fed mice. No significant difference was found in the body weight, food intake, Fat mass (FM), or fat-free mass (FFM) (Fig. 1A–D) between the HFD and BM-V groups at baseline (week 0). Energy intake at week 2 and week 3 was significantly lower in the BM-V animals when compared with HFD animals, and there were no significant difference between HFD and BM groups after week 3 of initiate treatment. From weeks 5 to 12 of initiated intervention, body weight was significantly lower in the BM-V group than in HFD animals. The FM was significantly increased and FFM were significantly decreased in the HFD group during 12-week study, but these parameters between week 0 and week 12 were not significantly altered in BM-V mice. FM was significantly higher at week 12 than week 0 in HFD group (P < 0.01). FM was significantly lower and FFM were significantly higher in BM-V mice in compared with HFD mice after the 12-week intervention (Fig. 1C and D). Actual food intake was no difference between HFD and BM-V groups (Fig. 1 E). Feed efficiency data in these animals show that FCR was much higher in the BM-V group than in the HFD from week 6 to week 12 (P < 0.05, P < 0.01 and P < 0.001, Fig. 1F).

**Figure 2:**
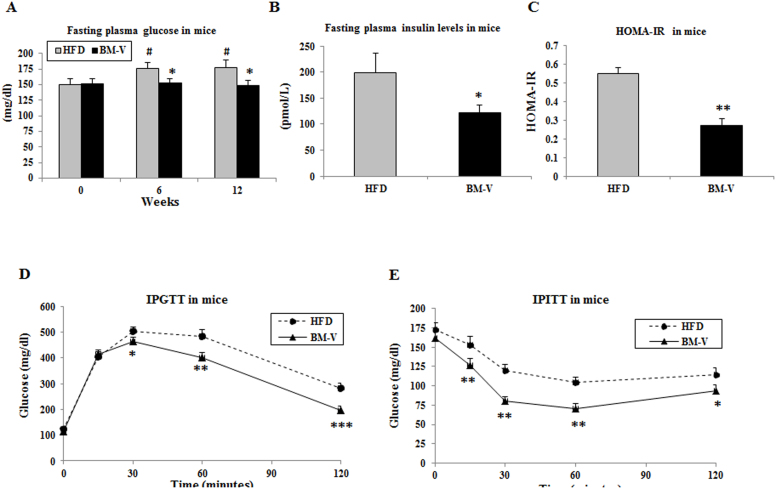
BM extracts improved glucose metabolism and insulin sensitivity in mice. Fasting plasma glucose levels (A) were measured at weeks 0, 6 and 12. Plasma insulin concentrations (B), HOMA-IR (C), IPGTT (D) and IPITT (E). Data are presented as mean ± SEM (n = 10/group). * P < 0.05, ** P < 0.01 and *** P < 0.001. BM-V vs. HFD group.

BM extract significantly improved glucose metabolism and enhanced insulin sensitivity in mice. No differences in plasma glucose concentrations were observed between HFD and BM-V groups at baseline. However, glucose levels significantly increased in the HFD groups from week 6 to week 12 (P < 0.05, Fig. 2A) when compared with week 0, but no significant changes were observed in BM-V group relative to its week 0. Plasma glucose concentrations were significantly lower in BM-V mice in comparison to HFD mice at week 6 and week 12 (P < 0.05, Fig. 2A). Plasma insulin concentration was significantly decreased, and insulin sensitivity (assessed by HOMA-IR) was significantly improved in BM-V group when compared with HFD group (P < 0.05 and P < 0.01, Fig. 2 B, C). IPGTT data show that glucose concentrations were significantly lower in BM-V than in HFD mice at 30, 60 and 120 min post glucose IP injection (P < 0.05, P < 0.01 and P < 0.001, Fig. 2 D). Glucose disappearance, as assessed by IPITT results, demonstrated that BM-V significantly enhanced the effect of insulin on glucose disposal (Fig. 2E).

**Figure 3:**
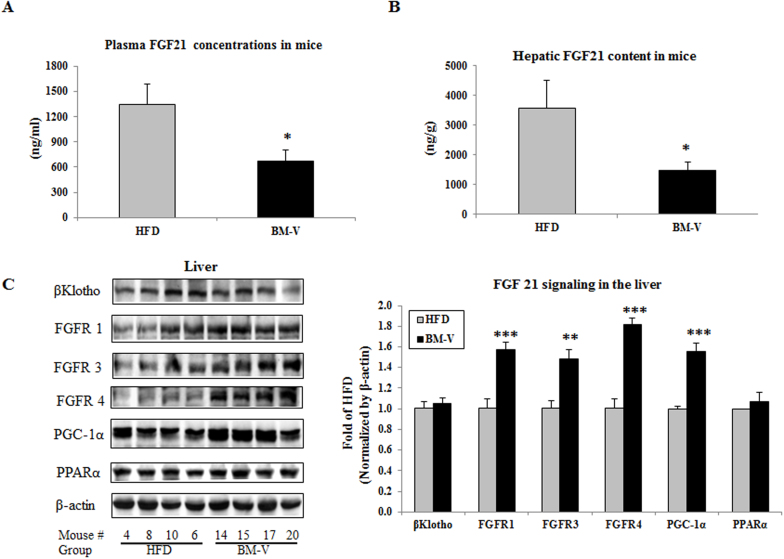
The effects of BM extracts on fasting plasma and liver FGF21 levels as well as FGF21 signaling in mice. FGF21 was measured using a mouse FGF21 ELISA kit from R & D Systems Inc (Minneapolis, MN). (A) Fasting plasma FGF21 concentrations. (B) Liver FGF21 content. Mean ± SEM (n = 10/group). (C) FGF21 signaling proteins were measured by Western blotting assay. Results were normalized by β-actin content. BM-V significantly increased FGFR1, FGFR3, FGFR4 and PGC-1α, slightly reduced PPARα, but did not affect b-Klotho protein abundance in comparison with HFD animals. Mean ± SEM (n = 10/group). * P < 0.05, ** P < 0.01, and *** P < 0.001, BM-V group vs. HFD group. # P < 0.05, glucose concentrations at week 6 or week 12 vs. week 0 in HFD animals. The blots in this figure are cropped. The full length blots are supplied in the supplementary information.

BM-V extract altered liver FGF 21 signaling in mice. Fasting plasma FGF21 levels and hepatic FGF21 content were significantly reduced in the BM-V group in comparison with HFD group (P < 0.05, Fig. 3A–B). Moreover, BM-V supplementation significantly increased hepatic FGFR1 (57%), FGFR3 (49%), FGFR4 (82%) and PGC-1α (55%) content (P < 0.001, P < 0.01, P < 0.001 and P < 0.01, respectively), slightly increased βKlotho (5%) and PPARα (7%) content when compared with HFD animals (Fig. 3C).

**Figure 4:**
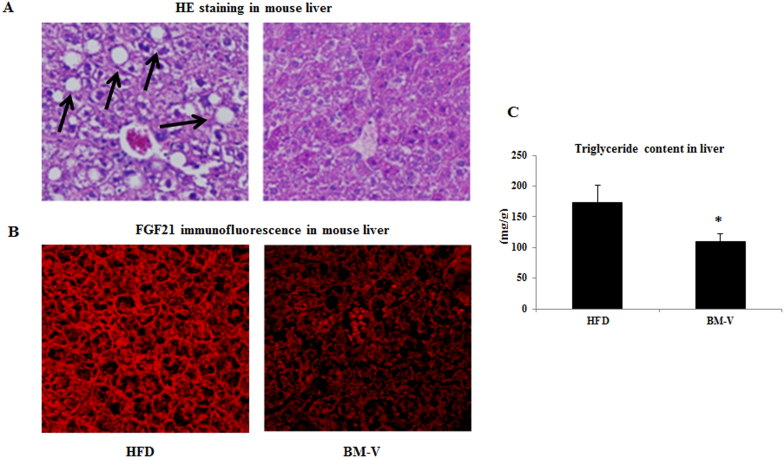
BM extracts reduce liver lipid and FGF21 content in mice. Hepatic histological and immunofluorescence microscope experiments were performed in the HFD and BM-V mice. (A) The morphological features of liver were evaluated by HE staining in the HFD-fed mice treated with or without BM-V extracts. Arrows denote hepatic macrovesicular steatosis. (B) Immunofluorescence image results of hepatic FGF21 in mice (original magnification × 200). (C) TG content was measured in lipid extracts from liver using a triglyceride assay kit. Results were normalized by protein concentration and represented as μmol/g protein. Mean ± SEM (n = 10/group). * P < 0.05, BM-V group vs. HFD group.

Morphological features of liver tissues were evaluated by H&E staining. The typical macrovesicular steatosis, hepatocellular ballooning, portal and lobular inflammatory cell infiltrations were observed in the HFD animals after 12 week of feeding (Figure 4A). Hepatic FGF21 content determined by Immunofluorescence microscopy was higher in HFD than in the BM-V group (Figure 4B). Supplementation with BM-V was shown to significantly reduce liver TG content in comparison with the HFD group (BM-V vs. HFD, P < 0.05, Figure 4C).

Plasma lipid profile analysis showed that fasting plasma TG, cholesterol and LDL-cholesterol concentrations were lower in the BM-V animals than in the HFD animals, but only plasma TG in the BM-V group was significantly lower (P < 0.05). There was no significant difference in plasma HDL-cholesterol levels between groups (Table 1).

**Figure 5:**
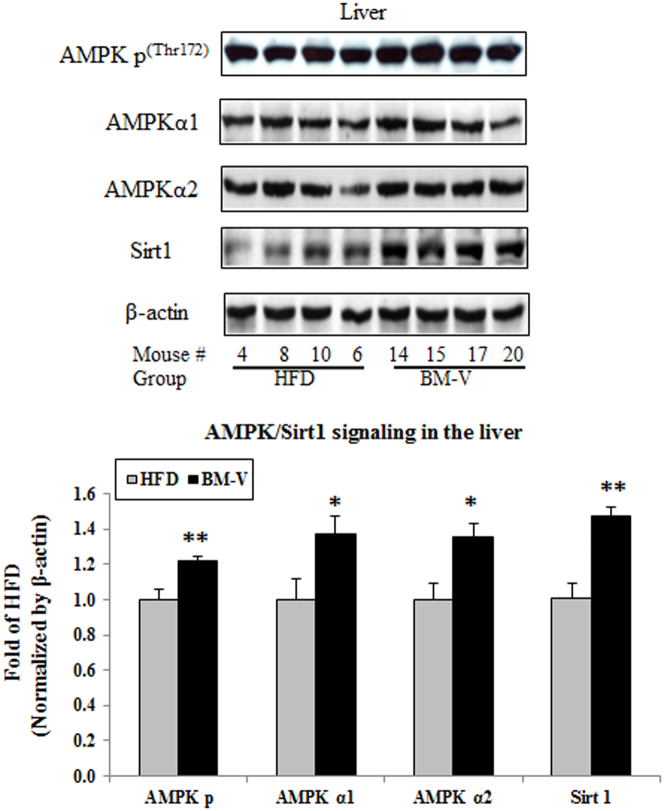
Effects of BM extracts on AMPK-Sirt1 signaling pathways in mice liver. Fifty μg of liver lysates was subjected to SDS-PAGEs, AMPK p, AMPK α1, AMPKα2 and Sirt1 were detected with corresponding specific antibodies. The results were normalized using β-actin as protein loading control. The data were represented as mean ± SEM (n = 10/group), * P < 0.05 and ** P < 0.01, BM-V vs. HFD group. The blots in this figure are cropped. The full length blots are supplied in the supplementary information.

Since AMPK-Sirt1 pathway may regulate FGF21 signaling, we determined AMPK-Sirt1 signaling proteins in the liver. The fold change of hepatic AMPK p content in BM-V vs. HFD group was 1.22 ± 0.09 vs. 1.00 ± 0.06 (n = 10, Mean ± SEM, P < 0.01), AMPKα1 abundance was 1.37 ± 0.10 vs. 1.00 ± 0.12 (P < 0.05), AMPK α2 was 1.36 ± 0.07 vs. 1.00 ± 0.09 (P < 0.05) and Sirt1 1.47 ± 0.05 vs. 1.00 ± 0.09 (P < 0.01), respectively. BM-V significantly increased the abundance of these proteins in the liver comparison with the HFD group (P < 0.05, Fig. 5).

## Discussion

In this study we assessed the effects of a BM extract on insulin sensitivity, liver fat content, and FGF21 and AMPK/Sirt-1 signaling in mice fed a high-fat diet. We observed that the BM-V extract significantly reduced body weight, fasting plasma glucose, insulin, and FGF21 concentration as well as liver FGF21 and TG content in mice fed a HFD. Furthermore, the extract improved plasma lipid profiles and enhanced insulin sensitivity when compared with the HFD group without significantly affecting food intake^25^. BM extract reduced the palatability of the diet as shown by the food intake at first three weeks of initiate intervention, which was significant lower in the BM-V group than in HFD group. However, these animals were able to adapt BM mixed diet quickly and consumed the same amount of food when compared with HFD after 3 weeks (Fig. 1B) and actual food intake (g/mouse/week) was slightly higher in BM mice than in HFD mice (Fig. 1E). Compared with regular 5%–10% dietary fiber supplementation experiments and caloric restriction studies i.e. 20%–30% lower food intake, HFD mixed with 1.2% BM extract seemed to not significantly affect the energy density of the diet in comparison with pure HFD. Therefore, the higher FCR in the BM-V group may contribute to either increased energy expenditure or reduced caloric absorption from the gut or both of them. Although the oxygen consumption rate in these mice were not measured, our data indicate that decreased adiposity in BM-supplemented rats may result from lower metabolic efficiency, a consequence of increased lipid oxidation and mitochondrial uncoupling^26^.

A major contributor to NAFLD may be insulin resistance, and a major contributor to insulin resistance is obesity, especially abdominal obesity^4^. In an in vitro study, BM extract reduced lipid accumulation during differentiation from pre-adipocyte to adipocyte, with a reduction in overall triglyceride of 32.4% after 72 hours compared with untreated control cells^27^. Recent study shows that aqueous extract of *Momordica charantia* seeds (MCSE) primarily regulated the insulin signaling pathway in muscles and adipose tissues with targeting insulin receptor (IR)^28^. Here, for the first time illustrated that the novel effects of BM-V on attenuated or reversed fatty accumulated in the liver of mouse on a HFD by modulating FGF21 signaling. FGF21 has potential insulin mimetic effects on lowering plasma glucose and liver lipid levels, suggesting that FGF21 may play a role in the pathogenesis of liver and whole-body insulin resistance in T2DM^17^. FGF21 injection in the brain increased energy expenditure and insulin sensitivity in obese rats^29^. On the other hand, clinical studies showed that plasma FGF21 levels were significantly higher in both insulin resistant obese and T2DM patients than in healthy subjects^7^,^29^,^30^. We observed that FGF21 resistance develops in mice fed a HFD (Plasma FGF21 levels in the HFD group were 4.7-fold higher than low-fat diet fed group, P < 0.001, unpublished data). Increase of FGF21 in the HFD mice may indicate that there is a compensative mechanism of the liver to secrete more FGF21 protein in the conditions of impaired FGF21 signaling. More recent studies reported that FGF21 levels were increased in association with obesity and NAFLD^31^. Thus, obesity is also linked to a FGF21- resistant condition^32^. In this study, we demonstrated that BM-V reduced plasma FGF21 levels and hepatic FGF21 content, and attenuated HFD-induced liver steatosis, while BM-V also significantly enhanced peripheral insulin sensitivity. The effects of BM extracts on reducing FGF21 levels may be contributed to enhance FGF21 signaling, and therefore, BM works more like as FGF21 “sensitizer” instead of agonist or antagonist of FGF21. Hyperinsulinemia, elevated FFA and glucose have also been noted to induce FGF21 expression in human studies^9^,^33^,^34^. BM extract robustly reduced hepatic FGF21 content in HFD-fed mice, but it remains to evaluate whether BM extract directly suppresses liver FGF21 expression without affecting PPARα transcription or indirectly inhibits FGF21 expression by blocking the effect of PPARα on FGF21 regulation. In vitro studies suggest that FGF21 initiates its action by activating a unique dual receptor complex consisting of a co-receptor βklotho and the tyrosine kinase FGFR^35^. βKlotho binds FGF21 and facilitates the activation of other FGFRs^36^. It is well documented that FGF21 is selective for FGFR1 isoform 1c, with varying reports of using isoforms 2c or 3c^37^,^38^. FGFR4 is dominant in mature hepatocytes and involved in the control of hepatic bile acid and lipid metabolism^39^. Recent study shows that FGF21 binds FGFR1 with much higher affinity than FGFR4 in presence of βKlotho; while FGF19 binds both FGFR1 and FGFR4 in presence of βKlotho with comparable affinity^40^. The increase of FGFR1, FGFR3 and FGFR4 protein abundance may reflect a negative feed-back regulatory mechanism of FGF21 on its receptors. BM supplementation resulted in reduction of liver FGF21expression and induction of its receptor expression in the HFD fed mice, indicating that BM extract enhances FGF21 signaling, by which attenuates HFD-induced insulin resistance and hepatic steatosis.

Skeletal muscle and adipose tissues secrete FGF21, but hepatocytes contribute greatly to FGF21 levels in response to free fatty acid (FFA) stimulation of a PPARα/RXR dimeric complex^37^,^38^. Other studies have reported that there was a positive correlation between FGF21 concentration, insulin levels, and body mass index (BMI) in human clinical and animal studies^5^,^31^. A study in Chinese subjects found a positive association of plasma FGF21 with circulating triglycerides, total cholesterol and gamma-glutamyltransferase, but not insulin sensitivity^41^. FFAs also increased circulating FGF-21, while insulin had little effect under physiological conditions. These observations may help explain the apparent paradox of increased FGF21 levels in obesity, insulin resistance, and starvation^38^. Consistent with Tan's report that BM extracts increased activity of AMPK, a key pathway mediating glucose uptake and fatty acid oxidation^23^, we observed that BM-V significantly increased AMPK phosphorylation, AMPKα1 and AMPKα2 protein abundance when compared with the HFD mice. Furthermore, BM-V significantly increased hepatic Sirt1 protein abundance in comparison with the HFD. The findings that BM extract enhanced FGF21 and AMPK-Sirt1 signaling pathways support the notion that FGF21 regulates mitochondrial activity and enhances oxidative capacity through an AMPK-SIRT1-PGC1alpha-dependent mechanism in adipocytes^12^.

Taken together, BM extract supplementation greatly enhances insulin sensitivity, reverses HFD-induced liver damage, and significantly reduces plasma FGF21 levels and body fat mass in mice fed a HFD. This study suggests that the favorable effects of a BM extract on increasing insulin sensitivity and attenuating hepatic steatosis may be mediated by enhanced FGF21 and AMPK-Sirt1 signaling. Therefore, a BM extract presents a potential botanical target for the development of new therapeutic alternatives to treat NAFLD, obesity, and diabetes.

## Methods

A commercially available BM extract powder from alcohol extraction of fresh bitter melon was tested. The BM extract was kindly provided by Verdure Sciences Inc, Noblesville, IN (BM-V) containing 3.3% of momordicosides A, F1, G, K, and L. The extract was well characterized by HPLC/LC-MS analysis with standards of momordicosides listed above obtained from the University of Mississippi (USA, supplemental data, table 1). All other reagents, unless mentioned, were purchased from Sigma-Aldrich (St. Louis, MO).

All animal experiments were performed according to a protocol approved by the Institutional Animal Care and Use Committee of Pennington Biomedical Research Center. Thirty 5-week-old male C57BL/6J mice were ordered from Charles River Laboratories, Inc (Wilmington, MA) and maintained at constant temperature and humidity (21 ± 2°C with humidity 65–75%) with a 12:12-h light-dark cycle. Mice were housed two/cage, labeled with ear punch, and allowed access to water and food ad libitum.

Mice were randomly divided into two groups; high-fat diet control (HFD) and HFD supplementation with bitter melon extract (BM-V). Animals were fed a HFD containing 58% of energy from fat (D-12331) purchased from Research Diets Inc. (New Brunswick, NJ). BM was administered by incorporating the extract into the high-fat diet at a dose of 1.2% (W/W). Briefly, 500 g of high-fat diet and 6 g of BM extract powder were mixed with a food processor (Cuisinart DLC-2014, Sears, Hoffman Estates, IL); food dye was added to monitor HFD completely mixed with BM extract. Then the mixed diet was stored in −20°C for the feeding experiments. Chosen 1.2% BM extract was based on our previous study^24^. Food intake and body weight were recorded weekly. Fasting glucose was measured at weeks 0, 6, and 12 of the study respectively. Body composition was determined (described in the methods) at weeks 0 and 11. Fasting plasma insulin, FGF21 levels, and lipid profiles were measured at week 12. At the end of the study, the mice were euthanized. The liver and other tissues were dissected and snap frozen in the liquid nitrogen and stored at −80°C for future measurements.

### Blood chemistry and hormone analysis

After 4 hours of fasting, blood samples were collected by tail stick. Plasma glucose levels were measured by a colorimetric hexokinase glucose assay (Sigma Diagnostics, St Louis, MO). Plasma insulin levels were determined by mouse insulin enzyme-linked immunosorbent assay (ELISA) kits (Millipore Co. Billerica, MA). Homeostasis model assessment –insulin resistance (HOMA-IR) was calculated using the following formula: HOMA-IR = [I_0_ (μU/ml) × G_0_ (mmol/liter)]/22.5^42^. Plasma FGF21 was measured by mouse FGF-21 ELISA Kits according to the manufacturer's instructions (R & D Systems, Minneapolis, MN). Intra-assay and inter-assay CVs of FGF21 were 4.5% and 6.1%, respectively. FGF21 quality control result was 278 pg/ml (range 191–319 pg/ml).

### Body composition measurement

Body composition for all animals was measured using a Minispec TD-NMR Spectrometer (Bruker Optics, TX)^43^. Total fat mass (FM) and fat free mass (FFM) were recorded.

### Feed conversion ratio (FCR)

FCR is a measure of an animal's efficiency in converting feed mass into increased body mass. It was calculated at weeks 2, 4, 6, 8′ and 12 as the ratio of feed intake to gain in body weight^44^,^45^.

Intraperitoneal glucose tolerance testing (IPGTT) and intraperitoneal insulin tolerance testing (IPITT) were measured at weeks 10 and 11 respectively. IPGTT was performed after overnight fast, before and after mice were intraperitoneal injected glucose at dose 1 gram/kg body weight, blood glucose concentrations were measured at 0, 15, 30, 60 and 120 min using glucose strips described as below. For IPITT, after 4 h fast mice were IP injected insulin at 0.75 U/kg body weight^43^. Blood glucose concentrations were measured from the tail vein at time 0 (baseline) or 15, 30, 60 and 120 minutes after insulin injection using the Freestyle blood glucose monitoring system (Thera Sense, Phoenix, AZ).

### Plasma lipid profile analysis

Fasting plasma TG concentrations were assessed using a TG reagent kit from Eagle Diagnostics Inc (DeSoto, TX). Plasma cholesterol levels were measured with a cholesterol assay kit from BioVision Inc (Mountain View, CA). HDL-cholesterol level was determined by phosphotungstic acid and magnesium chloride precipitation method^46^.

### Liver FGF21 content assessment

Liver tissues (~25 mg) were added to ten volumes of homogenization buffer (w/v), minced with scissors in Eppendorf microcentrifuge tubes, and homogenized by micro-homogenizer^43^. Tubes were centrifuged at 8000 × g for 5 min. 50 μl of supernatant was assayed using a FGF21 ELISA kit as described above.

### Liver lipid extract for TG measurement

Liver lipid extracts were prepared by the Folch procedure^47^. Briefly, liver tissues (about 25 mg) were added to five volumes of PBS (w/v), minced with scissors in Eppendorf microcentrifuge tube, and homogenized by sonication. The liver lysates were added to ten volumes of an extract solvent containing chloroform and methanol in the ratio of 2:1 (v/v). After vortexing, the tubes were centrifuged at 5000 × g for 10 min. Aliquots of 100 μl were removed from the bottom of the tube, transferred to a new tube and dried under nitrogen gas. After adding 100 μl of PBS to the tube, 10 μl of mixture was taken to measure TG content using a TG assay kit (DeSoto, TX). The results were normalized by protein concentration.

### Histological studies in the liver

The sections of liver tissues from the center of the largest liver lobes were fixed in 10% buffered formaldehyde, and then embedded in paraffin. A 5 μm-thick section cut from a paraffin-embedded block was stained with hematoxylin and eosin (HE staining). All specimens were observed and photomicrographed using an Olympus 1X71 inverted microscope and Olympus PP72 camera, Olympus America Inc (Melville, NY).

### Immunofluorescence microscopy

Immunofluorescence for FGF21 was performed on liver slides. Briefly, the sections of liver tissues from the center of the largest liver lobes were fixed in 10% buffered formaldehyde, and then embedded in paraffin. A 5 μm-thick section cut was taken from the paraffin-embedded block. Slides were dewaxed using xylene, rehydrated with an alcohol gradient, and then incubated with a 1% Citrate buffer in 0.1% Triton X100, wash with PBS solution for 8 minutes to unmask antigen. A blocking solution containing 10% normal goat serum was incubated with the sample overnight at 4°C. Samples were incubated with a monoclonal anti-FGF21 (1:250) for 60 min at room temperature, and with Alexa Fluor 594-conjugated goat anti-mouse IgG (1:300 dilution; Invitrogen, Carlsbad, CA) for 60 min at room temperature. After sealing the slides, images were obtained on a Zeiss 510 Axiophot microscope equipped with a Nikon digital camera and processed using Metamorph imaging software, version 6.1 (Universal Imaging).

### Western blotting analysis

Liver lysates were prepared by homogenization in buffer A (25 mM HEPES, pH 7.4, 1% Nonidet P-40 (NP-40), 137 mM NaCl, 1 mM PMSF, 10 μg/ml aprotinin, 1 μg/ml pepstatin, 5 μg/ml leupeptin) using a PRO 200 homogenizer (PRO scientific, Oxford, CT). The samples were centrifuged at 14,000 g for 20 minutes at 4°C and protein concentrations of the supernatants were determined by Bio-Rad protein assay kit (Bio-Rad laboratories, Inc. Hercules, CA). Supernatants (50 μg) were resolved by 8% or 12% SDS-PAGE and subjected to immunoblotting. The protein abundance was detected with antibodies against FGF21 (R & D Systems, Minneapolis, MN), FGFR1, AMPK p^(Thr172)^, AMPK α1, AMPKα2, PPARα, and PGC-1α (Millipore, Billerica, MA), FGFR3 (Bioworld Technology, Inc, Louis Park, MN), βklotho and FGFR4 (Santa Cruz, CA), and β-actin (Affinity Bioreagents, Golden, CO) using Chemiluminescence Reagent Plus (PerkinElmer Life Science, Boston, MA), and quantified via densitometer. All the proteins were normalized to β-actin. Spectra™ multicolor broad range protein ladder (10–260 kDa, Pierce Biotech, Rockford, IL) was used as reference for the molecular weight of interesting protein.

### Statistical analysis

SAS univariate procedure with the normal option and the QQ plot statement was conducted to test normality of the data. Results were expressed as mean ± SEM. Comparisons between groups were determined by Student t-test. P values < 0.05 were considered statistically significant.

## Author Contributions

Z.W. designed the study; X.Z. and Y.Y. researched data. Z.W. performed statistical analysis and wrote this manuscript. D.R. reviewed it. B.E. purified BM extract. Z.W. has primary responsibility for final content. All authors reviewed and approved the manuscript.

## Supplementary Material

Supplementary Information Table 1

Supplementary Information Supplementary Dataset 1

Supplementary Information Supplementary Dataset 2

Supplementary Information Supplementary Dataset 1

## Figures and Tables

**Table 1:** Plasma lipid profile in mice after treated with BM-v for 12 weeks

Groups	HFD (mg/dL)	BM-V
Cholesterol	246 ± 22	222 ± 15
Triglyceride	173 ± 27	119 ± 16*
LDL-cholesterol	153 ± 21	118 ± 14
HDL-cholesterol	78 ± 1.8	80 ± 0.8

Mean ± SEM (n = 10/group);

*P < 0.05, BM-V group vs. HFD group.
