# Supplementary material for: Bitter melon extract attenuating hepatic steatosis may be mediated by FGF21 and AMPK/Sirt1 signaling in mice
Source: Sci Rep. 2013 Nov 5;3:3142. doi: 10.1038/srep03142 (PMC3912441; doi:10.1038/srep03142)
Supplement: Supplementary Information — Table 1 [file srep03142-s1.doc]

Bitter melon extract attenuating hepatic steatosis may be mediated by FGF21 and AMPK/Sirt1 signaling in mice

Yongmei Yu^1^ , Xian H Zhang^1^, Blake Ebersole^2^, David Ribnicky ^3^ and Zhong Q. Wang^1*^

*^1^Nutrition and Diabetes Research Laboratory, Pennington Biomedical Research Center, LSU System. Baton Rouge, LA 70808*

*^2^Verdure Sciences, 1250 Conner St, Noblesville, IN 46060*

*^3^Dept. of Plant Biology and Path., Rutgers University, New Brunswick, New Jersey 08901*

*Address for correspondence: Zhong Q Wang, M.D

SREP-13-03631A

|  | Table 1 Plasma lipid profile in mice after treated with BM-v for 12 weeks | | | | | |  |
| --- | --- | --- | --- | --- | --- | --- | --- |
|  | Groups | HFD | BM-V |  | |  | |
|  |  | (mg/dL) |  |  | |  | |
|  | Cholesterol | 246 ± 22 | 222 ± 15 |  | |  | |
|  | Triglyceride | 173 ± 27 | 119 ± 16* |  | |  | |
|  | LDL-cholesterol | 153 ± 21 | 118 ± 14 |  | |  | |
|  | HDL-cholesterol | 78 ± 1.8 | 80 ± 0.8 |  | |  | |
|  |  |  |  |  | |  | |
|  | Mean ± SEM (n=10/group), * P<0.05, BM-V group vs. HFD group | | | |  | | |
|  |  |  |  |  | |  | |
