# Supplementary material for: Bitter melon extract attenuating hepatic steatosis may be mediated by FGF21 and AMPK/Sirt1 signaling in mice
Source: Sci Rep. 2013 Nov 5;3:3142. doi: 10.1038/srep03142 (PMC3912441; doi:10.1038/srep03142)
Supplement: Supplementary Information — Supplementary Dataset 1 [file srep03142-s4.pdf]

Bitter melon extract attenuating hepatic steatosis may be mediated by FGF21 and AMPK/Sirt1 signaling in mice

Yongmei Yu[1], Xian H Zhang[1], Blake Ebersole[2], David Ribnicky[3] and Zhong Q. Wang[1*]

[1]*Nutrition and Diabetes Research Laboratory, Pennington Biomedical Research Center, LSU System. Baton Rouge, LA 70808*

[2]*Verdure Sciences, 1250 Conner St, Noblesville, IN 46060*

[3]*Dept. of Plant Biology and Path., Rutgers University, New Brunswick, New Jersey 08901*

*Address for correspondence: Zhong Q Wang, M.D

SREP-13-03631A

Table 1 The components of BM-V extract analyzed using a HPLC/LC-MS

Momordicosides	A	F1	G	K	L	Total
BM-V	0.48%	0.58%	0.27%	0.14%	1.84%	3.30%
(Verdure MCA015EPPH)						

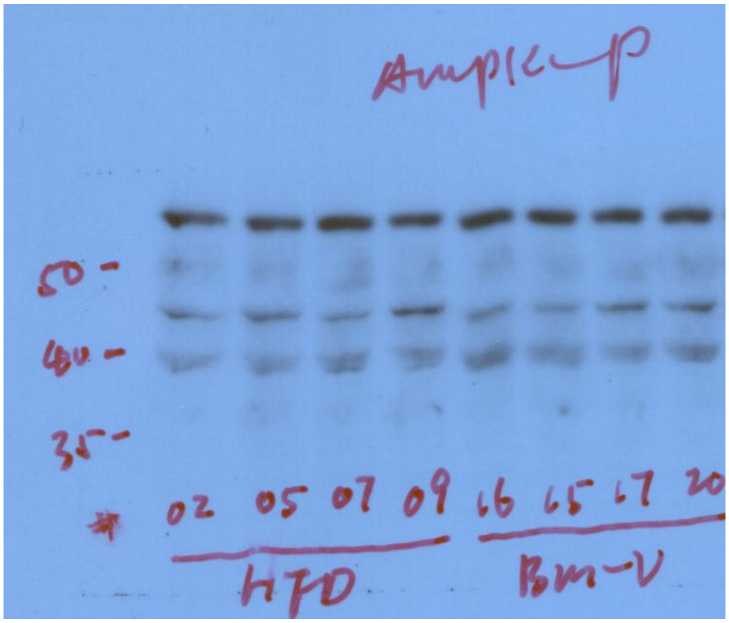

AMPK p

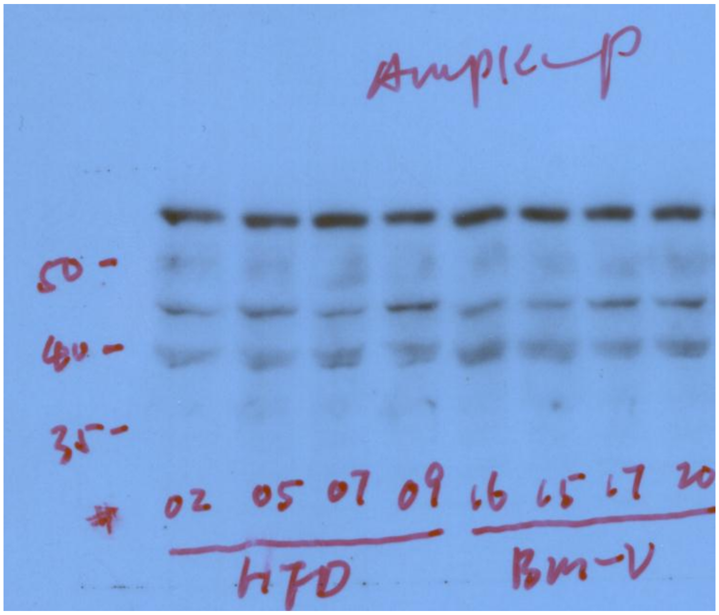

AMPKα1

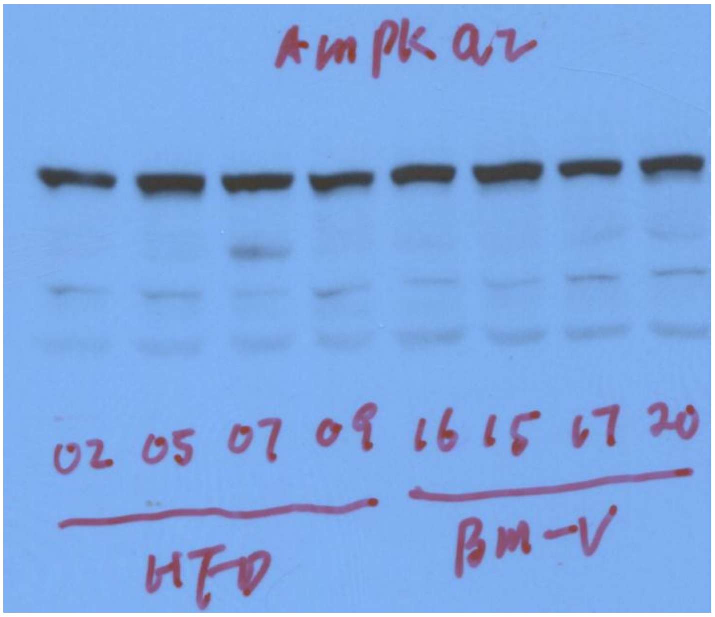

AMPKα2

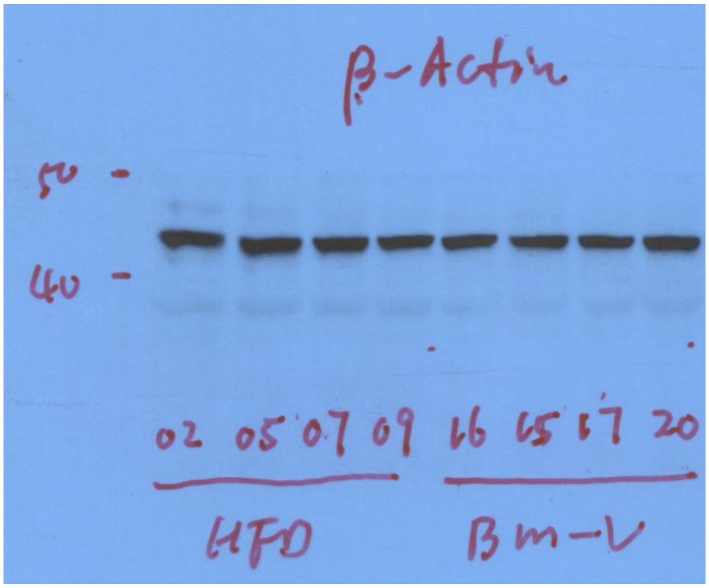

B-Actin1

Figure 5. Effects of BM extracts on AMPK-Sirt1 signaling pathways in mice liver. Fifty µg of liver lysates was subjected to SDS-PAGEs, AMPK p, AMPK α1, AMPKα2 and Sirt1 were detected with corresponding specific antibodies. The results were normalized using β-actin as protein loading control. The data were represented as mean ± SEM (n=10/group), * P< 0.05,   BM-V vs. HFD group.

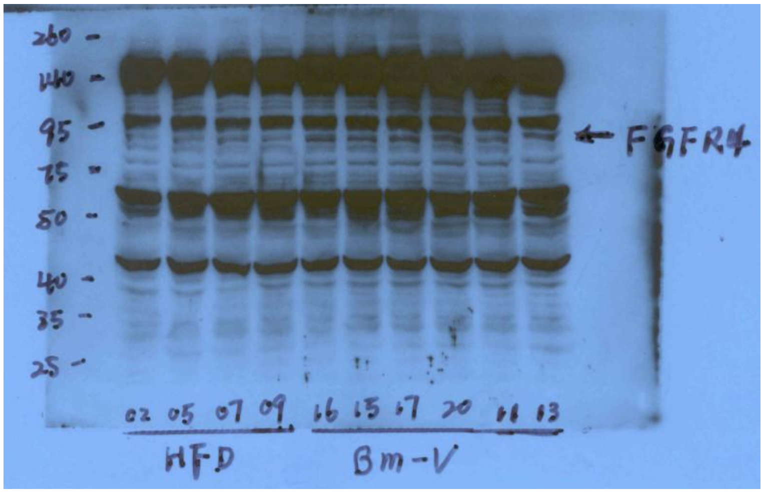

FGFR4

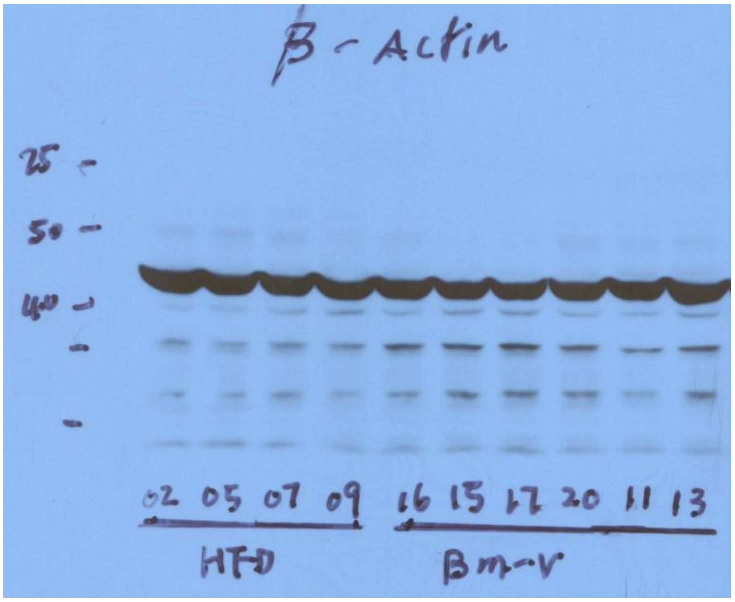

B-Actin2

Figure 3. The effects of BM extracts on fasting plasma and liver FGF21 levels as well as FGF21 signaling in mice. FGF21 was measured using a mouse FGF21 ELISA kit from R & D Systems Inc (Minneapolis, MN). (A) Fasting plasma FGF21 concentrations. (B) Liver FGF21 content. Mean ± SEM (n=10/group). (C) FGF21 signaling proteins were measured by Western blotting assay. Results were normalized by β-actin content. BM-V significantly increased FGFR1, FGFR3, FGFR4 and PGC-1α, slightly reduced PPARα, but did not affect b-Klotho protein abundance in comparison with HFD animals. Mean ± SEM (n=10/group). * $P < 0.05$, ** $P<0.01$, and *** $P <0.001$, BM-V group vs. HFD group. # $P<0.05$, glucose concentrations at week 6 or week 12 vs. week 0 in HFD animals.
